# Influence of Climate on Soil and Wine Bacterial Diversity on a Vineyard in a Non-traditional Wine Region in Argentina

**DOI:** 10.3389/fmicb.2021.726384

**Published:** 2021-08-12

**Authors:** Gabriel A. Rivas, Andrea C. Guillade, Liliana C. Semorile, Lucrecia Delfederico

**Affiliations:** ^1^Laboratorio de Microbiología Molecular, Departamento de Ciencia y Tecnología, Instituto de Microbiología Básica y Aplicada (IMBA), Universidad Nacional de Quilmes (UNQ), Bernal, Argentina; ^2^National Scientific and Technical Research Council Argentina (CONICET), Bernal, Argentina

**Keywords:** climate conditions, vineyard management, Malbec wine, amplicon sequencing, bacterial diversity

## Abstract

Argentina is the fifth world-wide wine producer, with an area of emerging importance in the Southwest of Buenos Aires Province, where climatic conditions are rather challenging. We studied the variations in soil and wine bacterial diversity through three consecutive vintages, and how climatic conditions affected said diversity. During the years of our study there were two harsh climatic events, a prolonged drought that extended over two vegetative periods, and an unseasonable spring frost in 2017. We found that the bacterial diversity reacted to these climatic events, given that there was a shift in the taxa exclusive to soil and wine, and shared by both, through time. Our results show a core of microorganisms in soil as well as in wine, belonging to different phyla that are conserved across the vintage years. A trend to an enrichment in *Actinobacteria* was detected in soil samples, whereas a high relative abundance of the *Acetobacteraceae* family and a scarcity of Lactic Acid Bacteria (LAB) were detected in the wine samples. We believe our results contribute to a better understanding of the impact of climatic conditions on the soil and wine microbiota, and can provide vintners with valuable knowledge for improving their wine production.

## Introduction

Argentina is ranked as the fifth wine producer after Italy, France, Spain and the United States, accounting for 4.15% (10.8 mhl) of the world’s wine production [International Organisation of Vine and Wine (OIV), 2020]. Although most of the traditional wine-producing regions are located along the Andes Mountains range, new vineyards have been recently established in Buenos Aires Province, in the transitional zone between the Pampeana and Espinal phytogeographic regions (Cabrera, 1976). Vintners have chosen this area because of its semiarid climate, the constant but moderate winds and the somewhat poor soil quality, favorable characteristics for wine grape culture. Although the region accounts for only 0.16% of the wine produced in Argentina [Instituto Nacional de Vitivinicultura (INV), 2020], it is a thriving activity of great cultural and economic value, providing wines of a unique *terroir*.

Macro climatic characteristics such as temperature, rainfall patterns and winds, can modify agricultural productivity either directly or indirectly (Liu et al., 2019), due to the influence of these factors on several viticultural features, such as the harvest dates, grape maturation (effect of temperature, radiation, and carbon dioxide), and vine pests. These viticultural variables can in turn impact winemaking by affecting fruit quality, sugar content and alcohol concentration, influencing changes in the microbiota and modifying acidity, potassium concentration and pH (De Orduna, 2010; Berbegal et al., 2019). Harvest date and the impact on grape maturation can increase the growth of spoilage microorganisms, the risks of starvation during the fermentation process, the release of toxic compounds, and alter the oxidation of volatile compounds (Campbell et al., 2016; Cook and Wolkovich, 2016). On the other hand, the increase in ethanol concentration due to the improvement in sugar content can increase the probability of sluggishness during the alcoholic fermentation, thus altering the organoleptic characteristics by reducing the perception of volatile compounds and increasing the astringency. Finally, higher pH allows the growth of other undesired microbes and produces changes in wine flavor, color, and aroma (Berbegal et al., 2019; Drappier et al., 2019).

Next Generation Sequencing (NGS) techniques are often used to better characterize the microbial diversity of complex ecosystems that play a key role in wine grapes culture (*Vitis vinifera*), wine production and quality (Liu et al., 2019). The microbial diversity has been identified as an important factor in the determination of the *terroir* (product identity) (Bokulich et al., 2014), along with the varietal, agricultural practices, soil texture, biogeographic location, and climatic conditions (Bokulich et al., 2013; Zarraonaindia et al., 2015; Belda et al., 2017; Liu et al., 2019). The bacterial group of greatest oenological relevance is lactic acid bacteria (LAB), responsible for decarboxylating malic into lactic acid during malolactic fermentation (MLF) and contributing to the sensory characteristics (flavor, aroma, and color) that give typicality to the final product (Campbell-Sills et al., 2017).

The structure of microbial communities and its persistence through time has been shown to be determined, at least in part, by the micro and mesoclimatic conditions, as well as the characteristics of the soil and vineyard (reviewed in Liu et al., 2019). Furthermore, the use of fertilizer and/or compost applications can modify the relative abundances of bacterial groups (Calleja-Cervantes et al., 2015; Morgan et al., 2017). Lapsansky et al., 2016 highlighted the concept of the memory of the soil, referred to soil’s resilience, in the sense of responding to and may recover from the stresses imposed by human activities or a changing climate. Certain agricultural practices directly affect the soil health, which involves complex physicochemical and microbiological parameters. However, through agricultural practices that ensure a sustainable management, it is possible to restore the soil health (Gabbarini et al., 2021).

Only recently have researchers begun assessing the diversity of microbial communities associated with Argentine vineyards by using NGS (Vega-Avila et al., 2015; Oyuela Aguilar et al., 2020), and they all have focused on the traditional wine regions in the country.

Given the novelty of vineyards as an agro-productive activity in Buenos Aires Province, the aim of this study was to assess the impact of climatic conditions over three consecutive harvests (2017–2019) on the bacterial communities associated with the soil and wine in a Malbec plot within a vineyard of this area. We believe our results can provide vintners with valuable knowledge for improving their wine production in the face of challenging conditions.

## Materials and Methods

### Winery and Vineyard Management

Saldungaray winery is located at a strategic site in a non-traditional wine-producing region in the Southwest of Buenos Aires Province, Argentina (38°12′54.5″S 61°46′36.3″W, 194 m.a.s.l., Supplementary Figure 1), corresponding to a transitional zone between the Pampeana and Espinal phytogeographic regions (Cabrera, 1976). The winery has been productive since 2003, with a total planted surface of 20 ha which include the varieties Malbec, Merlot, Chardonnay, Cabernet Sauvignon, Sauvignon Blanc, Tempranillo, and Cabernet Franc. At the beginning of our study, which covered Malbec plots from 2017 to 2019, the vines were 8 years old, and the plantation grid had a 2-m distance between rows and 1-m distance between vines, which were trained in a vertical shoot position. The pruning system is bilateral cordon de Royat, with the soil between rows covered with native grass. Surface water is provided by a drip irrigation system based on groundwater from an aquifer. A fan standing in one corner of the plantation protects the vineyard, along with the drip irrigation system, from frost damage. Pest and disease management involves soil applied herbicides (glyphosate acid Round-up^®^), as well as dithiocarbamate (Mancozeb^®^, Ziram^®^, and Zineb^®^) and phthalimide (Captan^®^, Folpet^®^) fungicides.

For winemaking, the winery employs a manual selection of the grapes and fermentation tanks made of steel or concrete to obtain young wines. The process begins with a cold pre-fermentation for 48–72 h and involves the use of the commercial yeast Uvaferm BC^®^ (Lallemand Inc., Montréal, QC, Canada) as a starter in alcoholic fermentation, which takes place in concrete tanks for about 10 days, followed by pressing, and then malolactic fermentation for 25–40 days. It is noteworthy that no malolactic bacteria starter has ever been used in this winery, therefore whenever malolactic fermentation occurs, it is spontaneous. Lastly, it is important to mention that the winemakers have reported a slowdown in the malolactic fermentation throughout the years.

### Analysis of the Local Climatic Conditions

The area where the Saldungaray winery is located has been termed “the Argentine arid diagonal,” a strip of land receiving scarce rainfall that stretches from Northern Peru to the Patagonian Atlantic coastline (Campo et al., 2009). The climate is temperate semi-arid, with marked seasonality in rainfalls, which occur mainly in spring and fall (Campo et al., 2009) and moderate to strong winds all year round, but particularly intense from late spring to mid-summer (Campo et al., 2011), predominantly West winds. The area is also characterized by extreme meteorological events like hail, frost, droughts, and floods, that take place in cyclic patterns (Campo et al., 2011). These climatic characteristics result in limited groundwater availability for irrigation systems.

Climatic data was obtained from the Sistema de Información y Gestión Agrometeorológica, INTA database^1^ for the years 2011–2019. Maximum and minimum daily temperatures were analyzed to obtain base-line monthly averages for the decade. Statistical analyses of temperatures were carried out for the vegetative periods 2016–2017, 2017–2018, and 2018–2019, considering that in the Southern hemisphere, the vegetative year begins with the leaf fall in April (early autumn) and ends with the harvest, during March (late summer). We used one-way ANOVA tests with Tuckey *post hoc* contrasts (Sokal and Rohlf, 1995) to compare daily temperatures per month among the three vegetative periods. Monthly rainfall and potential evapotranspiration were used to build hydrological balances for every vegetative period within the 2011–2019 decade, following the methodology by Thornthwaite and Mather (1957), considering a field capacity of 100 mm (Casado et al., 2007). Statistical analyses were carried out using Statistix 8 (Analytical Software, 2003).

### Soil Characterization

To determine the soil type in the Malbec plots, bulk soil samples were analyzed by the Laboratory of the Instituto de Suelos of the Instituto Nacional de Tecnología Agropecuaria (INTA, Hurlingham, Buenos Aires, Argentina) by triplicate. Furthermore, soil samples for each vintage under study (2017–2019) were sent by triplicate to the “Laboratorio Inagroy -Tecnoagro SRL,” CABA, Argentina, for physico-chemical characterization. The parameters estimated were pH, electric conductivity (dS/m), water saturation (% v/w), organic carbon (% w/w), organic material (% w/w), organic Nitrogen (% w/w), C/N ratio, assimilable phosphorus (% w/w), and extractable sulfur (ppm). The specific methodologies used for estimating each parameter are given in Table 1.

**TABLE 1:** Chemical and texture characterization of the Saldungaray’s soil from the three consecutive vintages analyzed (2017, 2018, and 2019).

**Methodology**	**Identification**	**2017**	**Soil quality**	**2018**	**Soil quality**	**2019**	**Soil quality**
Potentiometric (IRAM—SAGPyA 29574)	pH 1:2.5 water	^*A*^7.83 ± 0.15	Slightly alkaline	^*A*^7.57 ± 0.40	Slightly alkaline	^*B*^8.27 ± 0.12	Moderately alkaline
Conductimetric (IRAM-SAGPyA 29579)	Electric conductivity (dS/m)	0.90 ± 0.20	Not saline	0.60 ± 0.10	Not saline	0.87 ± 0.31	Not saline
Calculation (IRAM-SAGPyA 29578: 2009)	Water saturation (% v/w)	46.67 ± 0.58	–	47.00 ± 1.00	–	47.00 ± 0.00	–
Standard environmental quality—Soil quality (IRAM-SAGPyA 29571- 3: 2016)	Organic carbon (% w/w)	0.82 ± 0.07	–	1.00 ± 0.25	–	0.85 ± 0.05	–
Calculation (according to Read J W, Ridgell R H (1921))	Organic material (% w/w)	1.42 ± 0.12	Very poor	1.72 ± 0.44	Poor	1.46 ± 0.09	Very poor
Modified Kjeldahl method (IRAM-SAGPyA 29572:2019)	Organic nitrogen (% w/w)	0.08 ± 0.01	Very poor	0.09 ± 0.02	Very poor	0.08 ± 0.00	Very poor
Calculation	Relation C/N (s/u)	9.87 ± 1.46	−	10.53 ± 0.60	–	10.00 ± 0.61	–
Bray Kurtz method (IRAM-SAGPyA 29570-1:2010)	Assimilable phosphorus (% w/w)	6.23 ± 1.46	Low	11.63 ± 6.73	Low	6.93 ± 0.40	Low
	Extractable sulfur (S-SO4) (ppm)	14.10 ± 4.23	Medium	8.73 ± 2.93	Low	11.50 ± 4.48	Medium
Texture characterization	Soil texture determination (IRAM-SAGyP 29581)	Clay < 2 μm (% w/w)				20.27 ± 2.31	
		Total slime 2–50 μm (% w/w)				31.43 ± 1.14	
		Very fine sand-I 50–100 μm (% w/w)				15.80 ± 2.23	
		Fine sand 100–250 μm (% w/w)				10.17 ± 1.22	
		Medium sand 250–500 μm (% w/w)				0.50 ± 0.10	
		Gross sand 500–1000 μm (% w/w)				0.37 ± 0.12	
		Very gross sand 1–2 mm (% w/w)				0.13 ± 0.06	

*Statistical analysis was performed for each parameter (Kruskal-Wallis with Mann Whitney tests) and significant differences are indicated with different letters (p < 0.05).*

### Sample Collection and Preparation

A total of 25 samples were obtained: six soil samples for each vintage (2017, 2018, and 2019), three Malbec wine samples from each vintage (2017 and 2019), and one mixed must sample from the 2018 vintage.

#### Soil Samples

Soil samples were collected from three vines in the same row, taken adjacent to each vine (within a 40-cm radius) at 20–30 cm depth, and skipping three plants between each collection. The process was repeated on two other rows, with one row in between. The samples were randomly mixed, obtaining two biological triplicates for each vintage (*n* = 6 for each vintage).

Approximately 150–250 g of soil was placed in sterile stomacher bags (Nasco WHIRL-PAK^®^, United States), which were labeled and divided for chemical and microbiological analysis and stored separately to avoid cross contamination. Once in the laboratory, samples were stored at −20°C until processing.

#### Wine Samples

The wine samples for the 2017 and 2019 vintages consisted of grape must (24 h after destemming and crushing), fermentation stage one (FS1, day six of the fermentation process, i.e., alcoholic fermentation in development) and fermentation stage two (FS2, day 13 of the fermentation process, i.e., alcoholic fermentation finished) of the Malbec variety. The final wine had 13.4 and 13.8% ethanol respectively, with total SO_2_ 50 mg/L each. Additionally, pH and L-acid malic was evaluated for each sample studied. On the other hand, for the 2018 vintage, the winemakers reported a great loss in productivity during the months prior to harvest (which will be addressed in later sections), resulting in insufficient yield to produce wines of each variety. Consequently, only one grape must sample could be obtained, comprised of a mixture of the varieties Pinot Noir, Chardonnay, Sauvignon, and Malbec; this must was used as a base for the sparkling wine that the winery routinely produces.

### DNA Extraction

DNA from soil samples was extracted with the FastDNA Spin Kit for Soil (MP Biomedicals, LLC, Solon, OH, United States), following the supplier’s instructions. Regarding the wine samples, since the DNA extraction process was more challenging, the protocol had to be modified to obtain quality genomic DNA and to accomplish the quality and integrity criteria established for NGS techniques. Briefly, an aliquot of 35 mL of each wine sample was centrifuged for 15 min at 8,000 rpm, and the pellets were washed twice with Tris-EDTA buffer (TE) (20: 2 mM) and then resuspended in PBS. Then, 1 mL was added to an Eppendorf tube with glass beads, with the following lysis conditions: MT Lysis Buffer, two cycles of 1:30 min in a bead beater, and incubation of 2 min on ice between each cycle.

The DNA obtained was visualized on a 1% agarose gel, stained with ethidium bromide (0.5 mg/mL), to check for integrity. In addition, absorbances at 260, 280, and 230 nm (NanoDrop^®^ ND-1000 Thermo Fisher Scientific) were measured as an additional quality parameter to determine the total DNA concentrations and 260/280 and 260/230 absorbance ratios of each sample. Those that exceeded a concentration of 20 ng/μL and had a 260/280 ratio in the range of 1.7–2.1 and a 260/230 ratio in the range of 1.5–2.1 were selected.

### Sequencing

A NGS technique (amplicon sequencing) was used to identify partial sequences of the 16S rRNA gene of bacteria. The genomic DNA samples were sent to Macrogen Korea (Seoul, Rep. of Korea), where the amplicon libraries were prepared (Herculase II Fusion DNA Polymerase Nextera XT Index Kit V2). The hypervariable region V3-V4 of the 16S rRNA gene, obtained using primers Bakt_341F: 5′-CCTACGGGNGGCWGCAG-3′ and Bakt_805R: 5′-GACTACHVGGGTATCTAATCC-3′, were sequenced by Illumina (MiSeq). Sequences paired-end with 301 bp of length were obtained. The sequence data was deposited at NCBI (bioproject PRJNA742427).

### Sequence Analysis

Raw sequences fastq files were demultiplexed, chimeric sequences were filtered, and sequence ends were treated to remove low-quality regions, using QIIME2 (Callahan et al., 2016). Also, mitochondrial and chloroplast DNA were filtered (McDonald et al., 2012a, b). The OTUs table was obtained using DADA2 (Callahan et al., 2016). The fidelity in the reading depth was evaluated by means of rarefaction curves (qiime diversity alpha-rarefaction of QIIME2). The variation across samples was normalized by rarefying to 780 reading depths to performance alfa (richness and Shannon index) and beta (Bray Curtis distances estimation) diversity without bias. Statistical analysis was performed using PERMANOVA (test pseudo-F) for beta diversity and a non-parametric Kruskal-Wallis test (Kruskal and Wallis, 1952) for alpha diversity.

For taxonomic analysis, the database Greengenes (v13) was used (DeSantis et al., 2006; McDonald et al., 2012a, b), and the setting of the classifier training was performed using our primers, sequences, and tables obtained previously. This process was performed on QIIME2 using “q2-feature-classifier” plugin (Pedregosa et al., 2011; Bokulich et al., 2018) and “qiime feature-classifier classify-sklearn”. Venn diagrams were built from presence/absence matrices and the graphics from these results were designed using Corel Draw 2020 software.

## Results

### The Region Often Suffers Harsh Climatic Events

**FIGURE 1:**
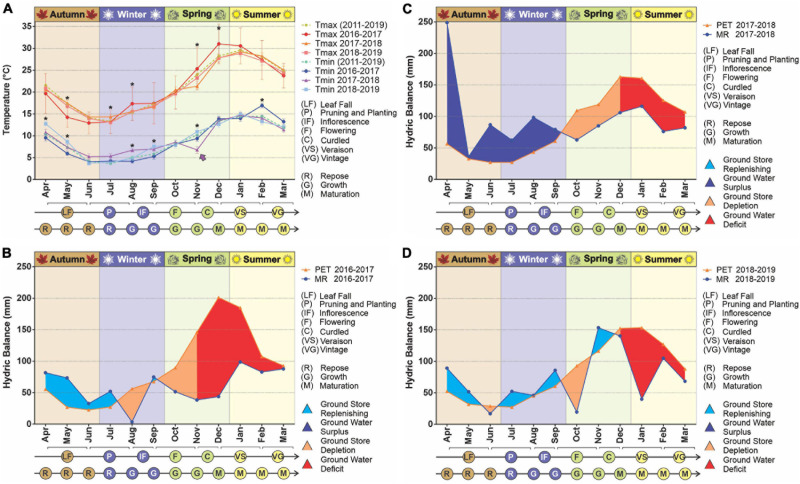
Climatic conditions in the years 2017–2019. The temperature variation was evaluated over time **(A)**. Statistical analyses were performed only for the three vegetative years under study; significant differences among years, for any given month, are indicated with an asterisk (*p* < 0.05). The decadic averages for minimum and maximum temperatures are added as a reference. The arrow indicates a frost, an unusual weather event for the season. The hydrologic balances were built for the years 2016–2017 **(B)**, 2017–2018 **(C)**, 2018–2019 **(D)**. The evaluated parameters were potential evapotranspiration (PET) and monthly rainfall (MR).

The temperatures in the study area, both minimum and maximum, varied significantly between the vegetative periods analyzed (Figure 1). At the beginning of the 2016–2017 vegetative period the maximum temperatures were lower than in the following years, with a hotter summer (*p* < 0.05). On the other hand, at the beginning of 2018 the minimum temperatures were slightly but significantly higher compared to the other periods, while for November 2017 (spring), a markedly lower temperature was recorded (*p* < 0.05), including an unseasonable frost. According to reports by the Saldungaray winery, this event had a vastly negative impact on the fruits that were in the process of growth/ripening, resulting in a loss of grape production, which prevented the individual elaboration of the different varietals (Malbec, Chardonnay, Pinot Noir, and Sauvignon).

Regarding ground water availability, from the hydrological balances built for the decade, we observed a pattern in which there is a recovery time, usually during the beginning of the vegetative period (April), even to the point of hydric excess, followed by a more or less extended period of ground water depletion, then water deficit (drought) until harvest time. This pattern was noticeable throughout the decade (Supplementary Figure 2) with the exception of the vegetative years 2012–2013 and 2013–2014, in which rainfall exceeded evapotranspiration in late summer. For the years studied, the longest drought period was 2016–2017, which began with the depletion of reserves in mid-July (winter) 2016, followed by a long period of water deficit from early November (mid-spring) until March 2017 (late summer). Conversely, at the beginning of the vegetative year 2017–2018, a great ground water surplus was registered followed an atypically abundant rainfall in April, which sustained the ground water reserve until mid-September (early spring). Finally, the vegetative period from 2018 to 2019 was characterized by less drastic differences in rainfall and evapotranspiration, resulting in a less pronounced period of water deficit (Figures 1B–D). These frequent water shortages led to lower yields, especially in the period 2015–2017. The year of lowest production was 2018, as a consequence of the unseasonable frost already mentioned (Supplementary Figure 2). Within the decade, 2016 was the worst year for the whole country, due to severe drought, partially recovered in 2017, and a remarkable recovery in 2018 (International Organisation of Vine and Wine (OIV), 2020).

### A Very Low Organic Carbon and Nitrogen Content, and a Slight Raising in pH, Characterized the Vineyard Soil Through the Years

The soil type in the Malbec plot was characterized as loamy, according to the relative proportions of lime, clay, and sand (USDA soil texture diagram). The physicochemical analysis showed an increase in the pH during the 2019 vintage (8.27), compared to the 2017 (7.83) and 2018 (7.57) vintages (*p* < 0.05), all in the range corresponding to mild-moderate/moderately alkaline soils, while the rest of the parameters analyzed remain without statistical differences. All the soil samples showed a poor to very poor organic material, carbon and nitrogen contents. Further details of soil physicochemical characteristics are shown in Table 1.

### Bacterial Diversity

Through the massive sequencing by Illumina (MiSeq) of the V3-V4 gene of the 16S rRNA gene, a total of 3924181 reads were obtained, with a high number of readings per sample (from 200087 to 113591). After depuration of the sequences (see section “Sequence Analysis” in “Materials and Methods”), the operative taxonomic units (OTU) were estimated for each sample, obtaining from 8,521 to 16,891 OTUs for the soil samples, and from 780 to 5,346 OTUs for the wine samples. The sequencing depth was evaluated through the analysis of the rarefaction curves, which showed a plateau that would indicate that an adequate sequencing depth was reached to perform the subsequent analyses (Supplementary Figure 3).

**FIGURE 2:**
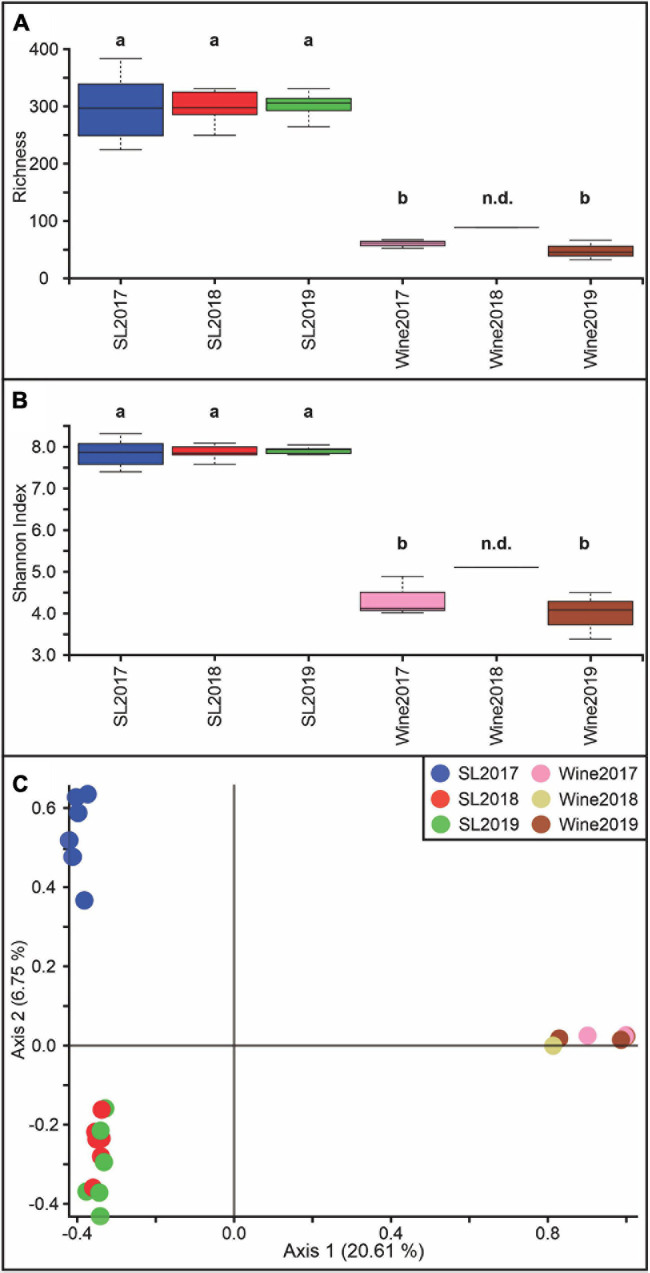
Bacterial diversity estimation at the Saldungaray vineyard for the years 2017, 2018, and 2019. For the soil (SL) and wine samples, Alpha diversity was estimated based on richness **(A)** and the Shannon index **(B)**. Different letters indicate statistical differences (*p* < 0.05). There was not enough data for the 2018 wine sample, therefore statistics remain non-determinate (n.d.). Principal coordinate analysis (PCoA) was performed to analyze the beta diversity at the Saldungaray vineyard based on Bray Curtis distances **(C)**.

The microbial diversity analysis showed that the internal richness was approximately 3 times lower (Figure 2A) in the wine samples than in the soil samples, while the Shannon index was 2 times lower (Figure 2B) throughout the years 2017, 2018, and 2019 (*p* < 0.05). Figure 2C shows a differential distribution on PCoA graphics for the soil-2017 sample, which would indicate a difference in the bacterial diversity with respect to the soil samples for the 2018 and 2019 vintages (*p* < 0.05). These two groups also exhibit significant differences regarding the wine samples (*p* < 0.05).

### The Soil Bacterial Community Structure Showed Annual Variations

**FIGURE 3:**
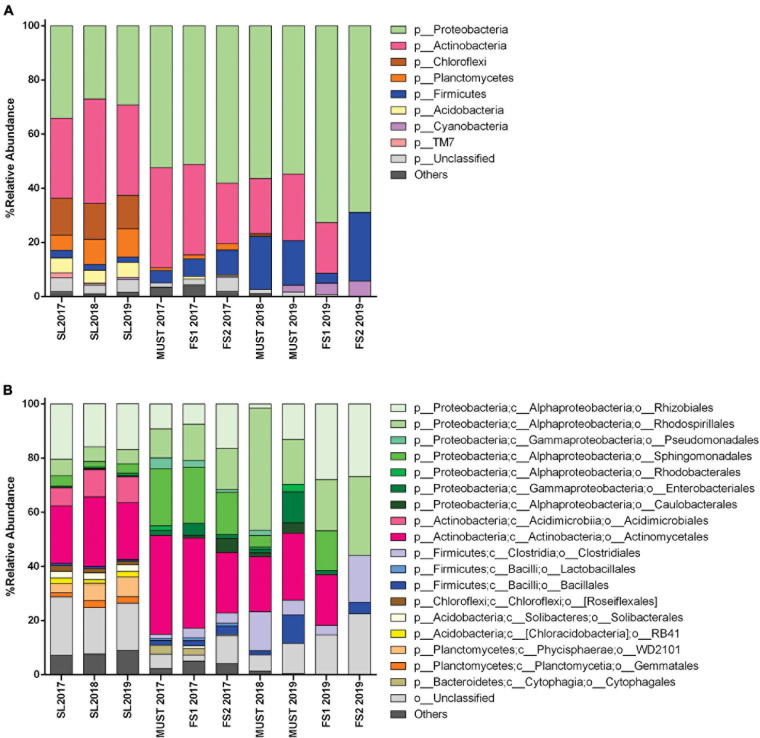
Bacterial community structure of soil (SL) and wine samples from the Saldungaray vineyard, at the phylum level **(A)**; the most abundant groups are shown at the order level **(B)**. Only OTUs exhibiting a relative abundance ≥ 0.5% are shown. In addition, the “others” category includes minority (<0.5%) OTUs.

The *Actinobacteria* and *Proteobacteria* were the dominant phyla (Figure 3A), and their frequency significantly changed thorough the sampling time. These phyla accounted for almost 60% of the total soil bacterial communities. The *Proteobacteria* were more abundant than the *Actinobacteria* in 2017 (34.1 and 29.5%, respectively); they decreased significantly in 2018 and increased slightly in 2019 (*p* < 0.05). The relative abundance of the *Actinobacteria* showed a significant increase between 2017 and 2018 (29.5–38.6%), slightly decreasing in 2019 (33.4%) (*p* < 0.05).

Other less abundant phyla found included *Chloroflexi, Planctomycetes, Firmicutes, Acidobacteria, Cyanobacteria*, and *Saccharibacteria (TM7). A*mong them, only *Planctomycetes* showed significant variations, increasing through the years from 5.5% in 2017 to 10.4% in 2019 (*p* < 0.05). On the other hand, the OTUs assigned to the phyla *Verrucomicrobia, Gemmatimonadetes*, and *Bacteroidetes*, often found in soil samples, were only present in some of the samples, or did not reach the minimum relative abundance (0.5%) required to be included in the analysis (Figure 3A). Although the 16S primers targeted mostly bacteria, archaeal sequences were also detected in soil samples from the three vintages (*Crenarchaeota* up to 0.38% and *Euryarchaeota* up to 0.14%). We assumed that the detection level of Archaea was artificially reduced due to the primers bias, thus, these OTUs were excluded for the analysis (Takahashi et al., 2014).

No archaeal sequences were detected in wine samples, and the role that these taxonomic groups could play in the winemaking process remains unknown.

**FIGURE 4:**
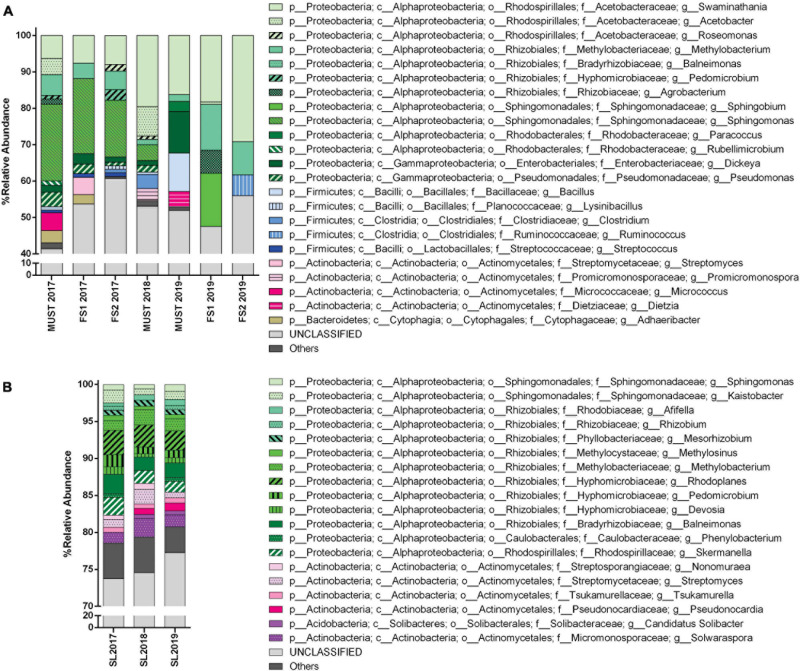
Bacterial community structure from the Saldungaray vineyard for the years 2017, 2018, and 2019, at the genus level. Bacterial diversity of wine **(A)** and soil samples **(B)**. Only OTUs exhibiting a relative abundance ≥ 0.5% are shown. In addition, the “others” category includes minority (<0.5%) OTUs.

At the order level, the taxonomic groups with the highest relative abundance in the soil samples were the *Actinomycetales* (21–25.7%), followed by the *Rhizobiales* (16–20.4%) and the *Acidimicrobiales* (6.7–10%). The significant increase in the *Actinobacteria* between 2017 and 2018 could be explained by the increase of the relative abundance of the orders *Actinomycetales*, which decreased again in 2019 (*p* < 0.05), and *Acidimicrobiales*, whose increase remain in 2019 (*p* < 0.05). In addition, the orders *Rhizobiales*, *Rhodospirillales*, and *Sphingomonadales*, belonging to the *Proteobacteria*, seem to be the main contributors to the reduction of this phylum between 2017 and 2018, and its partial recovery in 2019 (Figure 3B). The more complex nature of these orders, with several identified genera behaving differently across vintages (Figure 4B), could explain that these variations did not show statistical significance.

At the genus level, the number of OTUs from soil samples that could not be classified (unclassified group) was considerably high, around 80% (Figure 4A). The most dominant genera in these samples remain unclassified, belonging mainly to the *Actinomycetales*, particularly to the families *Streptomycetaceae, Nakamurellaceae*, and *Microbacteriaceae*, and their frequency did not change with the vintage year (Supplementary Figure 4). Of the genera belonging to the *Actinomycetales* that could be identified the most abundant was *Solwaraspora*, which increased from 1.5% in 2017 to 2.5% in 2018, and decreased to 1,6% in 2019; followed by *Streptomyces*, which was also more abundant in 2018 (2%), among others (Figure 4B).

A greater number of *Proteobacteria* than *Actinobacteria* OTUs could be identified to the genus level, mainly *Rhizobiales* (*Rhodoplanes, Balneimonas, Rhizobium*, and *Methylobacterium*), among others (Figure 4A); this could be attributed to a bias in the primers used.

### The Wine Microbiota Shows an Increase in the Relative Abundance of Acetic Acid Bacteria

Through the taxonomic identification of OTUs, we could assert that at the *phylum* level, *Proteobacteria* and *Actinobacteria* were the most abundant groups (except for the FS2-2019 sample, in which *Actinobacteria* were not recorded) (Figure 3A). These phyla accounted for almost 70–80% of the total bacterial community in the wine samples. *Firmicutes* is a relevant *phylum* in wine samples because it includes the order *Lactobacillales* -a technologically important group in wine, and it was abundant in the must samples 2018 (19.58%), and 2019 (16.43%), and in the FS2-2019 (25.36%) sample. However, from the estimation of bacterial diversity (Figure 3B) it is apparent that the *Lactobacillales* were absent from almost every sample, except for wine samples of 2017 vintage, in which they were recorded at a low relative abundance (1% or less). Moreover, at the order level, the most relevant groups were *Actinomycetales* (18–36%), *Rhodospirillales* (10–29%, and 45% for Must-2018), and *Rhizobiales* (7–27%, and 1.5% for Must-2018). Other relevant orders were the *Sphingomonadales* (14–21%, 4.35% Must-2018, but absent for Must-2019 and FS2-2019) and *Clostridiales* (1.5–5%, and 14–17% for Must 2018 and FS2-2019).

For wine samples, the number of OTUs that could not be classified (unclassified group) at the genus level increased considerably, up to 40–60% (Figure 4B). A high relative abundance was observed for the *Acetobacteraceae* family in all wine samples, particularly for the samples FS2-2019 (29%) and Must 2018 (45%); among this family we identified the genera *Swaminathania*, *Acetobacter*, and *Roseomonas*, acetic acid bacteria (AAB) which are potentially detrimental bacteria that may cause spoilage in wine (Supplementary Figure 4). In contrast, the only genus of lactic acid bacteria (LAB), detected exclusively in 2017 wine samples, was *Streptococcus*. Other relevant groups found were *Sphingomonas* in 2017 (15–21%) and 2018 (4%) wine samples, and *Methylobacterium* in 2017 (4–5%) and 2019 (9–12%, 1.8% for Must 2019) wine samples.

Additionally, the evolution of the winemaking process was also studied through the L-malic acid consumption and pH variations for 2017 and 2019 wine samples (Table 2). The 2018 wine sample was not considered because it was derived from a composite must containing several varieties, given that there was no Malbec vinification that year (see section “Wine Samples” in “Materials and Methods”). The initial L-malic acid concentration in must was higher in 2017 than in 2019, and so was the consumption (55% in 2017 vs. 40% in 2019). On the other hand, the pH decreased over time for 2017 wine samples, while for 2019 wine samples it was fluctuating.

**TABLE 2:** L-malic acid concentrations and pH evolution in the samples studied.

	**L-malic acid (gL^–1^)**	**pH**
Must 2017	1.57 ± 0.15	3.80
FS1 2017	1.03 ± 0.15	3.88
FS2 2017	0.70 ± 0.08	3.94
Must 2019	1.27 ± 0.03	3.88
FS1 2019	0.88 ± 0.05	3.78
FS2 2019	0.77 ± 0.05	3.89

### Shifts in Soil and Wine Microbiota Diversity Compared Through the Years

**FIGURE 5:**
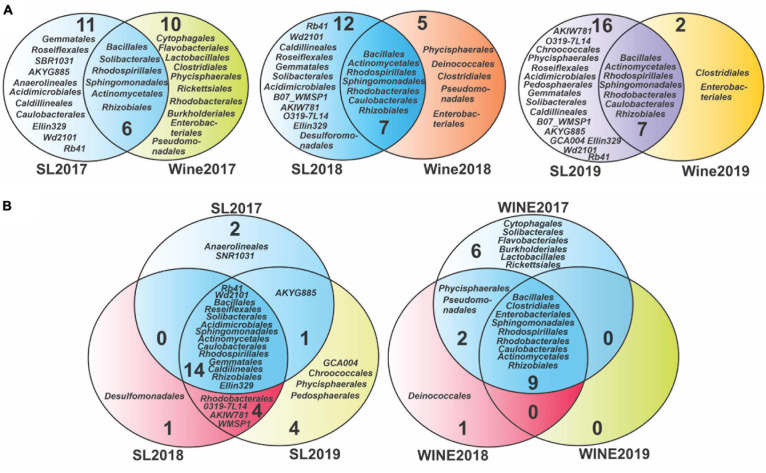
Venn diagram analysis for the bacterial diversity of Saldungaray vineyard at the order level. Soil-wine diversity relation **(A)**, soil-related diversity, and wine-related diversity according to vintage year **(B)**.

To evaluate the presence of bacterial taxonomic groups specific to a vintage year, the taxa identified were compared at the order level (Figure 5) and at genus level (Supplementary Figure 5). Venn diagrams were built at the order level to study the evolution of the taxonomic groups between the samples, which showed a core of microorganisms that persist throughout the years studied, consisting of 14 orders for the soil samples and 9 for the wine samples (Figure 5B). Those microorganisms that were recorded exclusively in each type of sample were also highlighted. For the soil, the number of specific taxonomic groups was variable, although it increased toward 2019: 2 were recorded for the year 2017 (*Anaerolineales* and *SNR1031*), 1 for the soil 2018 (*Desulfomonadales*), and 4 for the soil 2019 (*Chroococcales*, *Phycisphaerales*, *Pedosphaerales*, and *GCA004*). Conversely, a decrease in diversity was evidenced for wine samples, where we detected 6 unique orders in 2017 wine (*Lactobacillales*, *Rickettsiales*, *Solibacterales*, *Flavobacteriales*, *Cytophagales*, and *Burkholderiales*), 1 in 2018 (*Deinococcales*) and none in 2019.

Considering that the soil represents a substantial reservoir of biological diversity, special attention was paid to the microorganisms that were shared between these groups. We noticed that there is also a core of 6 orders that remain constant between soil and wine, during the three consecutive years, while for the years 2018 and 2019, the *Caulobacterales* were added, which in 2017 had only been present in soil samples (Figure 5A). On the other hand, the diversity of specific orders in soil samples increased from 11 (2017) to 16 (2019), whereas in wine samples it decreased from 10 (2017) to 2 (2019). The *Clostridiales* and the *Enterobacteriales* were the only specific orders found in the wine samples over the three consecutive years, while at the same time being absent from the soil samples.

## Discussion

### The Taxonomic Structure of Soil Microbial Communities Might Reflect Adaptations to Drought and Low Organic Carbon and Nitrogen Content

The Saldungaray winery is located in the southwest of Buenos Aires Province, currently considered as an emerging wine-producing region in Argentina. Although the semi-arid climate characteristic of the region is favorable in many senses for grapevine culture, the Argentine arid diagonal possesses other less desirable features, due to the action of opposing anticyclonic flows in the Southern hemisphere, as well as the influence of planet-wide phenomena such as El Niño Southern Oscillation. This results not only in cyclic periods of moderate to severe droughts, recorded since 1970 (Casado and Campo, 2019), but also in extreme weather events like hailstorms, frosts, severe thunderstorms and even the occasional tornado (Campo et al., 2009). A direct consequence of this climate is the unpredictable nature of groundwater availability, which in turn affects harvest yields in several of the most economically important crops in the province, namely wheat, maize, sunflower, and soy (Brescia et al., 1998).

During the years of our study, there were two climatic events of relevance. First and foremost, the drought that had begun in 2015 and extended until the 2017 harvest, and then the punctual event of the two unseasonable spring frosts in November 2017, which resulted in catastrophic yield losses for the 2018 harvest. Vintners reported that the frost fan was insufficient to prevent the damage caused by the extremely low temperature, so cold in fact that the water froze as soon as it left the irrigation sprinklers. Several frost events, albeit on a larger scale, occurred recently in France, in April (also during spring) 2021, affecting many French wine regions. The damage is still under evaluation and depends on the location of the plots, the local climatic conditions and even the early development of the buds.

Both the growth and health of crops and productivity are related to the associated microbiota. The dynamic interactions between grapevines and microorganisms can be modified because of climate factors or soil parameters, soil management practices, and plant age, among others (Vega-Avila et al., 2015; Bonfante et al., 2018; Berlanas et al., 2019; Vink et al., 2021).

This work is the first to study the bacterial diversity of the soil from a Malbec cultivar and the wine over three consecutive vintages (2017, 2018, and 2019) of a vineyard located in this strategic region. Other authors evaluated the diversity of microbial communities associated with conventionally managed vineyards in traditional winemaking regions that also suffer from water shortage in Argentina. They found the phyla *Proteobacteria* and *Actinobacteria* among the main taxonomic groups identified, although the *Proteobacteria* were in higher proportion (Vega-Avila et al., 2015; Oyuela Aguilar et al., 2020). Our results show the dominance of the same phyla across all samples, along with other less abundant phyla, which are typical for soils world-wide (Delgado-Baquerizo et al., 2018). The 2017 vintage, corresponding to the driest vegetative year, shows a slightly higher relative abundance of *Proteobacteria*, followed by a shift in the dominance of *Actinobacteria*, mostly *Actinomycetales* y *Acidimicrobiales*, in 2018 and 2019. The most obvious differences were observed in terms of beta diversity that grouped apart the samples from 2017. Other studies have also reported the prevalence of sequences ascribed to *Actinobacteria* in vineyard soils, some of them with an unusually high frequency (Novello et al., 2017; Vink et al., 2021). Many members of this phylum, particularly *Actinomycetales*, have slow growth rates, low nutritional requirements, and high affinity for complex molecules; furthermore, some of them have stress-resistance structures like thick cell walls or endospore formation capacity (Santos-Medellín et al., 2017). These combined features allow them to persist under drought or further stress conditions, such as the low availability of organic carbon in the vineyard soil under study. Several works have recorded significant enrichment in *Actinobacteria* in soil, rhizosphere and root-endophytes during droughts for different plants, and across a range of environments (Bouskill et al., 2013; Taketani et al., 2014; Chodak et al., 2015; Naylor et al., 2017).

Naylor et al. (2017) showed that drought provokes conserved shifts in bacterial community composition leading to a conservative response across a broad range of plant species. These results are somewhat consistent with ours, not only in the enrichment in *Actinobacteria*, but also in the observed core of soil microorganisms, conserved across the vintage years. This core includes various microorganisms, belonging to different phyla, although largely dominated by drought indicators. These were mostly *Actinobacteria*, (mostly *Actinomycetales*, that were the most abundant, including *Streptomycetaceae*, and *Acidimicrobiales*), and some members of *Proteobacteria* (mostly *Alphaproteobacteria* including *Rhizobiales* and *Rhodospirillales*) that can benefit nitrogen fixation, and could promote plant growth under drought or heat stress (Abd El-Daim et al., 2014; Taketani et al., 2014; Chodak et al., 2015; Naylor et al., 2017; Wipf et al., 2021).

We hypothesize that, whereas the microbiota from the soil may be well adapted to fluctuating and usually poor groundwater levels, and thus not much affected by the water deficit prior to the 2018 harvest, the above-ground microbiota, more exposed to climatic variations, was indeed affected by the punctual event, the frost that caused not only the lowest yield in the decade, but also a loss of microbial diversity in wine.

### The Wine Microbiota Is Less Diverse Over the Years, Enriched in AAB, and Scarce in LAB

The wine microbiota profiles are affected by the grapevine, including vintages (Oyuela Aguilar et al., 2020) and cultivar (Bokulich et al., 2014; Pinto et al., 2015; Wang et al., 2021). Alpha and beta diversities showed that the bacterial community of the wine samples was less diverse compared to the soil samples. Our results show that the contribution of soil bacteria to the structure of the wine microbial communities remains mostly unchanged over the vintage years, with a core of six to seven orders, which includes potentially detrimental AAB. This could imply that some external factor has forced the soil microbiota to adapt to new conditions and that these conditions were less and less compatible with the conditions or adaptations necessary for these microorganisms to proliferate in the wine. *Methylobacterium* was one of the four genera that we detected in 2017 in both soil and wine samples, and incidentally the only one shared between the 2019 soil and wine samples. These are endophytic bacteria that have been consistently isolated from wine samples and even cultured, although their specific role is yet to be understood (Bokulich et al., 2016). Although the soil serves as a primary reservoir for potential vine-associated bacteria, the other microbes absent in the soil and present in the wine could come from other sources, namely plant leaves, roots, vine trunk bark, and the winery setting (Bokulich et al., 2013; Zarraonaindia et al., 2015; Vitulo et al., 2019).

We detected LAB in all 2017 wine samples, but that was not the case for the 2019 samples even though we were able to obtain isolates of LAB after successive enrichment cultures from the 2019 samples (data not shown). We believe that LAB might have been in extremely low concentrations in the 2019 samples and the lack of detection might be due to a limitation in the sequencing technique employed.

During the fermentation process that involves the production of wine, a reduced microbial diversity is expected since only a select group of microorganisms will have the ability or adaptations necessary to survive the adverse conditions of wine (low pH, high temperature, deficiency of nutrients, presence of SO_2_, and high% ethanol). LABs are a group of great oenological and technological relevance because they are microorganisms that lead the fermentation processes, some of the most important species in wine being *Lactiplantibacillus plantarum* and *Oenococcus oeni*, widely recognized for leading malolactic fermentation (Lonvaud-Funel, 1999; Lerm et al., 2011; Valdes la Hens et al., 2015; Bravo-Ferrada et al., 2016; Brizuela et al., 2017).

Members of the *Acetobacteraceae* family could have come from the soil since, although they were found in a low relative abundance, they are one of the groups of bacteria colonizing the wine samples throughout the three years studied and during all the fermentation stages. Conversely, the absence of the *Lactobacillales* order in the soil could indicate that this niche would not be acting as a biological reservoir for this microbiological group.

The high relative abundance of the *Acetobacteraceae* family and the scarcity of LAB detected in the wine samples could be related to the already mentioned slowdown in MLF throughout the years, since the process is dependent on the strains and the concentration of LAB, while also sensitive to different concentrations of ethanol (Brizuela et al., 2017). L-malic acid consumption is desirable to be as high as possible, reaching a final concentration of less than 0.5 gL^–1^ (Bravo-Ferrada et al., 2016; Brizuela et al., 2017). Given that the last stage of fermentation of wine 2017 (FS2) studied reached a consumption of around 55% and a value greater than 0.5 gL^–1^ of L-malic acid, we could assume that this process was ongoing at the time of sampling. However, wine 2019 (FS2) had only 40% consumption as well as concentration of L-malic acid higher than 0.5 gL^–1^. We could attribute this low consumption of L-malic acid to those LAB that we could not detect but we were able to culture. Moreover, some non-Saccharomyces yeasts are also able to consume L-malic acid (Balmaseda et al., 2018).

In conclusion, we believe that our work provides valuable information for vintners in areas of comparable climatic conditions. We showed that the soil microbiota is largely adapted to these challenging conditions and can withstand even prolonged droughts. Since ground water deficit impacts on vineyard productivity, proper provisions regarding water management can help mitigate some of these effects. Moreover, it was evident that it was the punctual phenomenon of the unseasonal frost that most gravely affected productivity, but it also altered the presence and abundance of some of the soil bacterial groups that had been present hitherto. The microbial structure of the bacterial community of the wine seems to be also affected by climatic factors. This is relevant from the point of view of definition of terroir in the productive area, monitoring of microbiota under stressful conditions, and microbial ecology in general. Recent interactions with the vineyard owners have resulted in them adapting their practices according to the information provided by our technical reports regarding the bacterial composition of wine in the 2017 and 2019 vintages. They have eliminated the cold soaking prior to the fermentation process in order to shorten it and prevent proliferation of AAB. This is an encouraging example of a beneficial synergy between research and productivity. We hope to further assist the sector by expanding our understanding of the soil and wine microbiota, and the factors affecting this community.

## Data Availability Statement

The datasets presented in this study can be found in online repositories. The names of the repository/repositories and accession number(s) can be found below: https://www.ncbi.nlm.nih.gov/, PRJNA742427.

## Author Contributions

LS and LD contributed to the conception and design of the study. GR and AG performed the statistical analysis. AG performed the climatic analysis and revised English, redaction, and cohesion. GR performed the bioinformatic analysis, designed the figures and tables, and wrote the first draft of the manuscript. GR, AG and LD wrote sections of the manuscript. LS funded the study. All authors contribute to manuscript revision, read, and approved the submitted version.

## Conflict of Interest

The authors declare that the research was conducted in the absence of any commercial or financial relationships that could be construed as a potential conflict of interest.

## Publisher’s Note

All claims expressed in this article are solely those of the authors and do not necessarily represent those of their affiliated organizations, or those of the publisher, the editors and the reviewers. Any product that may be evaluated in this article, or claim that may be made by its manufacturer, is not guaranteed or endorsed by the publisher.

## Footnotes

1 Instituto Nacional de Tecnología Agropecuaria (INTA) databe. Sistema de Información y Gestión Agrometeorológica. http://siga2.inta.gov.ar/#/data (Accessed June 14, 2020).

## References

[B1] Abd El-Daim I. A. Bejai S. Meijer J. (2014). Improved heat stress tolerance of wheat seedlings by bacterial seed treatment. *Plant Soil* 379 337–350.

[B2] Analytical Software (2003). *Statistix 8 Users Manual.* Tallahassee: Analytical Software.

[B3] Balmaseda A. Bordons A. Reguant C. Bautista-Gallego J. (2018). Non-*Saccharomyces* in wine: Effect upon *Oenococcus oeni* and malolactic fermentation. *Front. Microbiol.* 9:534. 10.3389/fmicb.2018.00534 29628914 PMC5876288

[B4] Belda I. Zarraonaindia I. Perisin M. Palacios A. Acedo A. (2017). From vineyard soil to wine fermentation: microbiome approximations to explain the “terroir” concept. *Front. Microbiol.* 8:821. 10.3389/fmicb.2017.00821 28533770 PMC5420814

[B5] Berbegal C. Fragasso M. Russo P. Bimbo F. Grieco F. Spano G. (2019). Climate changes and food quality: The potential of microbial activities as mitigating strategies in the wine sector. *Fermentation* 5:85. 10.3390/fermentation5040085

[B6] Berlanas C. Berbegal M. Elena G. Laidani M. Cibriain J. F. Sagües A. (2019). The fungal and bacterial rhizosphere microbiome associated with grapevine rootstock genotypes in mature and young vineyards. *Front. Microbiol.* 10:1142. 10.3389/fmicb.2019.01142 31178845 PMC6538693

[B7] Bokulich N. A. Collins T. S. Masarweh C. Allen G. Heymann H. Ebeler S. E. (2016). Associations among wine grape microbiome, metabolome, and fermentation behavior suggest microbial contribution to regional wine characteristics. *MBio* 7:e0631–16. 10.1128/mBio.00631-16 27302757 PMC4959672

[B8] Bokulich N. A. Kaehler B. D. Rideout J. R. Dillon M. Bolyen E. Knight R. (2018). Optimizing taxonomic classification of marker-gene amplicon sequences with QIIME 2’s q2-feature-classifier plugin. *Microbiome* 6 1–17. 10.1186/s40168-018-0470-z 29773078 PMC5956843

[B9] Bokulich N. A. Ohta M. Richardson P. M. Mills D. A. (2013). Monitoring seasonal changes in winery-resident microbiota. *PloS one* 8:e66437. 10.1371/journal.pone.0066437 23840468 PMC3686677

[B10] Bokulich N. A. Thorngate J. H. Richardson P. M. Mills D. A. (2014). Microbial biogeography of wine grapes is conditioned by cultivar, vintage, and climate. *Proc. Natl. Acad. Sci.* 111 E139–E148. 10.1073/pnas.1317377110 24277822 PMC3890796

[B11] Bonfante A. Monaco E. Langella G. Mercogliano P. Bucchignani E. Manna P. (2018). A dynamic viticultural zoning to explore the resilience of terroir concept under climate change. *Sci. Total Environ.* 624 294–308. 10.1016/j.scitotenv.2017.12.035 29253777

[B12] Bouskill N. J. Lim H. C. Borglin S. Salve R. Wood T. E. Silver W. L. (2013). Pre-exposure to drought increases the resistance of tropical forest soil bacterial communities to extended drought. *ISME J.* 7 384–394. 10.1038/ismej.2012.113 23151641 PMC3554394

[B13] Bravo-Ferrada B. M. Hollmann A. Brizuela N. La Hens D. V. Tymczyszyn E. Semorile L. (2016). Growth and consumption of L-malic acid in wine-like medium by acclimated and non-acclimated cultures of Patagonian *Oenococcus oeni* strains. *Folia Microbiol.* 61 365–373. 10.1007/s12223-016-0446-y 26801155

[B14] Brescia V. Lema D. Parellada G. Documento D. T. (1998). *El fenómeno ENSO y la agricultura pampeana: impactos económicos en trigo, maíz, girasol y soja.* Argentina: Instituto Nacional de Tecnología Agropecuaria, Instituto de Economía y Sociología.

[B15] Brizuela N. S. Bravo-Ferrada B. M. La Hens D. V. Hollmann A. Delfederico L. Caballero A. (2017). Comparative vinification assays with selected patagonian strains of *Oenococcus oeni* and *Lactobacillus* plantarum. *LWT* 77 348–355. 10.1016/j.lwt.2016.11.023

[B16] Cabrera A. L. (1976). “Regiones fitogeográficas argentinas,” in *Enciclopedia argentina de agricultura y jardinería. Tomo 2.* 2a edición Edn, ed. En Kugler W. F. (Argentina: Acme. Buenos Aires), 1–85.

[B17] Callahan B. J. McMurdie P. J. Rosen M. J. Han A. W. Johnson A. J. Holmes S. P. (2016). DADA2: High resolution sample inference from amplicon data. *Nat. Methods* 13 581–583. 27214047 10.1038/nmeth.3869 PMC4927377

[B18] Calleja-Cervantes M. E. Menéndez S. Fernández-González A. J. Irigoyen I. Cibriain-Sabalza J. F. Toro N. (2015). Changes in soil nutrient content and bacterial community after 12 years of organic amendment application to a vineyard. *Eur. J. Soil Sci.* 66 802–812. 10.1111/ejss.12261

[B19] Campbell B. M. Vermeulen S. J. Aggarwal P. K. Corner-Dolloff C. Girvetz E. Loboguerrero A. M. (2016). Reducing risks to food security from climate change. *Global Food Security* 11 34–43. 10.1016/j.gfs.2016.06.002

[B20] Campbell-Sills H. El Khoury M. Gammacurta M. Miot-Sertier C. Dutilh L. Vestner J. (2017). Two different *Oenococcus oeni* lineages are associated to either red or white wines in Burgundy: genomics and metabolomics insights. *OENO One* 51 309–322. 10.20870/oeno-one.2017.51.4.1861

[B21] Campo A. Ramos M. B. Zapperi P. (2009). *Análisis de las variaciones anuales de precipitación en el suroeste bonaerense, Argentina.* Montevideo: XII Encuentro de Geógrafos de América Latina, 12.

[B22] Campo A. M. Gil V. Gentili J. O. Volonté A. Duval V. (2011). Inventario de eventos climáticos–meteorológicos extremos. Suroeste bonaerense (1995-2010). *Párrafos Geográficos* 10 102–115.

[B23] Casado A. L. Campo A. M. (2019). Extremos hidroclimáticos y recursos hídricos: estado de conocimiento en el suroeste bonaerense, Argentina. *Cuadernos Geograficos* 58 6–26.

[B24] Casado A. L. Gil V. Campo A. M. (2007). Consecuencias de la variación de la disponibilidad hídrica en la cuenca del arroyo El Belisario, Buenos Aires, Argentina. *Revista Huellas* 11 9–26.

[B25] Chodak M. Gołȩbiewski M. Morawska-Płoskonka J. Kuduk K. Niklińska M. (2015). Soil chemical properties affect the reaction of forest soil bacteria to drought and rewetting stress. *Ann. Microbiol.* 65 1627–1637. 26273241 10.1007/s13213-014-1002-0 PMC4529456

[B26] Cook B. I. Wolkovich E. M. (2016). Climate change decouples drought from early wine grape harvests in France. *Nat. Climate Change* 6 715–719. 10.1038/nclimate2960

[B27] De Orduna R. M. (2010). Climate change associated effects on grape and wine quality and production. *Food Res. Int.* 43 1844–1855. 10.1016/j.foodres.2010.05.001

[B28] Delgado-Baquerizo M. Oliverio A. M. Brewer T. E. Benavent-González A. Eldridge D. J. Bardgett R. D. (2018). A global atlas of the dominant bacteria found in soil. *Science* 359 320–325. 10.1126/science.aap9516 29348236

[B29] DeSantis T. Z. Hugenholtz P. Larsen N. Rojas M. Brodie E. L. Keller K. (2006). Greengenes, a chimera-checked 16S rRNA gene database and workbench compatible with ARB. *Appl. Environ. Microbiol.* 72 5069–5072. 10.1128/AEM.03006-05 16820507 PMC1489311

[B30] Drappier J. Thibon C. Rabot A. Geny-Denis L. (2019). Relationship between wine composition and temperature: Impact on Bordeaux wine typicity in the context of global warming. *Crit. Rev. Food Sci. Nutr.* 59 14–30. 10.1080/10408398.2017.1355776 29064726

[B31] Gabbarini L. A. Figuerola E. Frene J. P. Robledo N. B. Ibarbalz F. M. Babin D. (2021). Impacts of switching tillage to no-tillage and vice versa on soil structure, enzyme activities and prokaryotic community profiles in Argentinean semi-arid soils. *FEMS Microbiol. Ecol.* 97:fiab025. 10.1093/femsec/fiab025 33571359

[B32] Instituto Nacional de Vitivinicultura (INV) (2020). *Informe Anual de Cosecha y Elaboración.* Available online at: https://www.argentina.gob.ar/sites/default/files/informe_anual_de_cosecha_y_elaboracion_2020_1.pdf (accessed June 14, 2020).

[B33] International Organisation of Vine and Wine (OIV) (2020). Available online at: https://www.oiv.int/public/medias/7541/en-oiv-2020-world-wineproduction-first-estimates.pdf (accessed June 14, 2020).

[B34] Kruskal W. H. Wallis W. A. (1952). Use of ranks in one-criterion variance analysis. *J. Am. Stat. Assoc.* 47 583–621. 10.1080/01621459.1952.10483441

[B35] Lapsansky E. R. Milroy A. M. Andales M. J. Vivanco J. M. (2016). Soil memory as a potential mechanism for encouraging sustainable plant health and productivity. *Curr. Opin. Biotechnol.* 38 137–142. 10.1016/j.copbio.2016.01.014 26897653

[B36] Lerm E. Engelbrecht L. du Toit M. (2011). Selection and characterization of *Oenococcus oeni* and *Lactobacillus* plantarum South African wine isolates for use as malolactic fermentation starter cultures. *S. Afr. J. Enol. Vitic.* 32 280–295.

[B37] Liu D. Zhang P. Chen D. Howell K. (2019). From the vineyard to the winery: how microbial ecology drives regional distinctiveness of wine. *Front. Microbiol.* 10:2679. 10.3389/fmicb.2019.02679 31824462 PMC6880775

[B38] Lonvaud-Funel A. (1999). Lactic acid bacteria in the quality improvement and depreciation of wine. *Antonie van Leeuwenhoek* 76 317–331. 10.1023/A:1002088931106 10532386

[B39] McDonald D. Clemente J. C. Kuczynski J. Rideout J. R. Stombaugh J. Wendel D. (2012a). The Biological Observation Matrix (BIOM) format or: how I learned to stop worrying and love the ome-ome. *Gigascience* 1:7. 10.1186/2047-217X-1-7 23587224 PMC3626512

[B40] McDonald D. Price M. N. Goodrich J. Nawrocki E. P. DeSantis T. Z. Probst A. (2012b). An improved Greengenes taxonomy with explicit ranks for ecological and evolutionary analyses of bacteria and archaea. *ISME J.* 6 610–618. 10.1038/ismej.2011.139 22134646 PMC3280142

[B41] Morgan H. H. Du Toit M. Setati M. E. (2017). The grapevine and wine microbiome: insights from high-throughput amplicon sequencing. *Front. Microbiol.* 8:820. 10.3389/fmicb.2017.00820 28553266 PMC5425579

[B42] Naylor D. DeGraaf S. Purdom E. Coleman-Derr D. (2017). Drought and host selection influence bacterial community dynamics in the grass root microbiome. *ISME J.* 11 2691–2704. 10.1038/ismej.2017.118 28753209 PMC5702725

[B43] Novello G. Gamalero E. Bona E. Boatti L. Mignone F. Massa N. (2017). The rhizosphere bacterial microbiota of Vitis vinifera cv. Pinot Noir in an integrated pest management vineyard. *Front. Microbiol.* 8:1528. 10.3389/fmicb.2017.01528 28855895 PMC5557794

[B44] Oyuela Aguilar M. Gobbi A. Browne P. D. Ellegaard-Jensen L. Hansen L. H. Semorile L. (2020). Influence of vintage, geographic location and cultivar on the structure of microbial communities associated with the grapevine rhizosphere in vineyards of San Juan Province, Argentina. *PloS One* 15:e0243848. 10.1371/journal.pone.0243848 33315910 PMC7735631

[B45] Pedregosa F. Varoquaux G. Gramfort A. Michel V. Thirion B. Grisel O. (2011). Scikit-learn: Machine learning in Python. *J. Mach. Learn. Res.* 12 2825–2830.

[B46] Pinto C. Pinho D. Cardoso R. Custódio V. Fernandes J. Sousa S. (2015). Wine fermentation microbiome: a landscape from different Portuguese wine appellations. *Front. Microbiol.* 6:905. 10.3389/fmicb.2015.00905 26388852 PMC4555975

[B47] Santos-Medellín C. Edwards J. Liechty Z. Nguyen B. Sundaresan V. (2017). Drought stress results in a compartment-specific restructuring of the rice root-associated microbiomes. *MBio* 8:e0764–17. 10.1128/mBio.00764-17 PMC5516253 28720730

[B48] Sokal R. R. Rohlf F. J. (1995). *biometry.*

[B49] Takahashi S. Tomita J. Nishioka K. Hisada T. Nishijima M. (2014). Development of a prokaryotic universal primer for simultaneous analysis of Bacteria and Archaea using next-generation sequencing. *PloS One* 9:e105592. 10.1371/journal.pone.0105592 25144201 PMC4140814

[B50] Taketani R. G. Kavamura V. N. Mendes R. Melo I. S. (2014). Functional congruence of rhizosphere microbial communities associated to leguminous tree from B razilian semiarid region. *Environ. Microbiol. Rep.* 7 95–101. 10.1111/1758-2229.12187 25870877

[B51] Thornthwaite C. W. Mather J. R. (1957). *Instrucciones y tablas para el cómputo de la evapotranspiración potencial y el balance hídrico.* New Jersey: Instituto Tecnológico de Drexel, 68.

[B52] Valdes la Hens D. Bravo-Ferrada B. M. Delfederico L. Caballero A. C. Semorile L. C. (2015). Prevalence of *Lactobacillus plantarum* and *Oenococcus* oeni during spontaneous malolactic fermentation in Patagonian red wines revealed by polymerase chain reaction-denaturing gradient gel electrophoresis with two targeted genes. *Austral. J. Grape Wine Res.* 21 49–56. 10.1111/ajgw.12110

[B53] Vega-Avila A. D. Gumiere T. Andrade P. A. M. Lima-Perim J. E. Durrer A. Baigori M. (2015). Bacterial communities in the rhizosphere of Vitis vinifera L. cultivated under distinct agricultural practices in Argentina. *Antonie Van Leeuwenhoek* 107 575–588. 10.1007/s10482-014-0353-7 25527391

[B54] Vink S. N. Chrysargyris A. Tzortzakis N. Salles J. F. (2021). Bacterial community dynamics varies with soil management and irrigation practices in grapevines (Vitis vinifera L.). *Appl. Soil Ecol.* 158:103807. 10.1016/j.apsoil.2020.103807

[B55] Vitulo N. Lemos W. J. F. Jr. Calgaro M. Confalone M. Felis G. E. Zapparoli G. (2019). Bark and grape microbiome of Vitis vinifera: influence of geographic patterns and agronomic management on bacterial diversity. *Front. Microbiol.* 9:3203. 10.3389/fmicb.2018.03203 30671035 PMC6331396

[B56] Wang H. L. Hopfer H. Cockburn D. W. Wee J. (2021). Characterization of microbial dynamics and volatile metabolome changes during fermentation of chambourcin hybrid grapes from two Pennsylvania Regions. *Front. Microbiol.* 11:614278. 10.3389/fmicb.2020.614278 33505380 PMC7829364

[B57] Wipf H. M. L. Bùi T. N. Coleman-Derr D. (2021). Distinguishing between the impacts of heat and drought stress on the root microbiome of Sorghum bicolor. *Phytobio. J.* 5:52. 10.1094/PBIOMES-07-20-0052-R

[B58] Zarraonaindia I. Owens S. M. Weisenhorn P. West K. Hampton-Marcell J. Lax S. (2015). The soil microbiome influences grapevine-associated microbiota. *MBio* 6:e02527–14. 10.1128/mBio.02527-14 25805735 PMC4453523

