# Supplementary material for: Influence of Climate on Soil and Wine Bacterial Diversity on a Vineyard in a Non-traditional Wine Region in Argentina
Source: Front Microbiol. 2021 Aug 12;12:726384. doi: 10.3389/fmicb.2021.726384 (PMC8406854; doi:10.3389/fmicb.2021.726384)
Supplement: Supplementary file 1 [file Data_Sheet_1.pdf]

Supplementary Material:

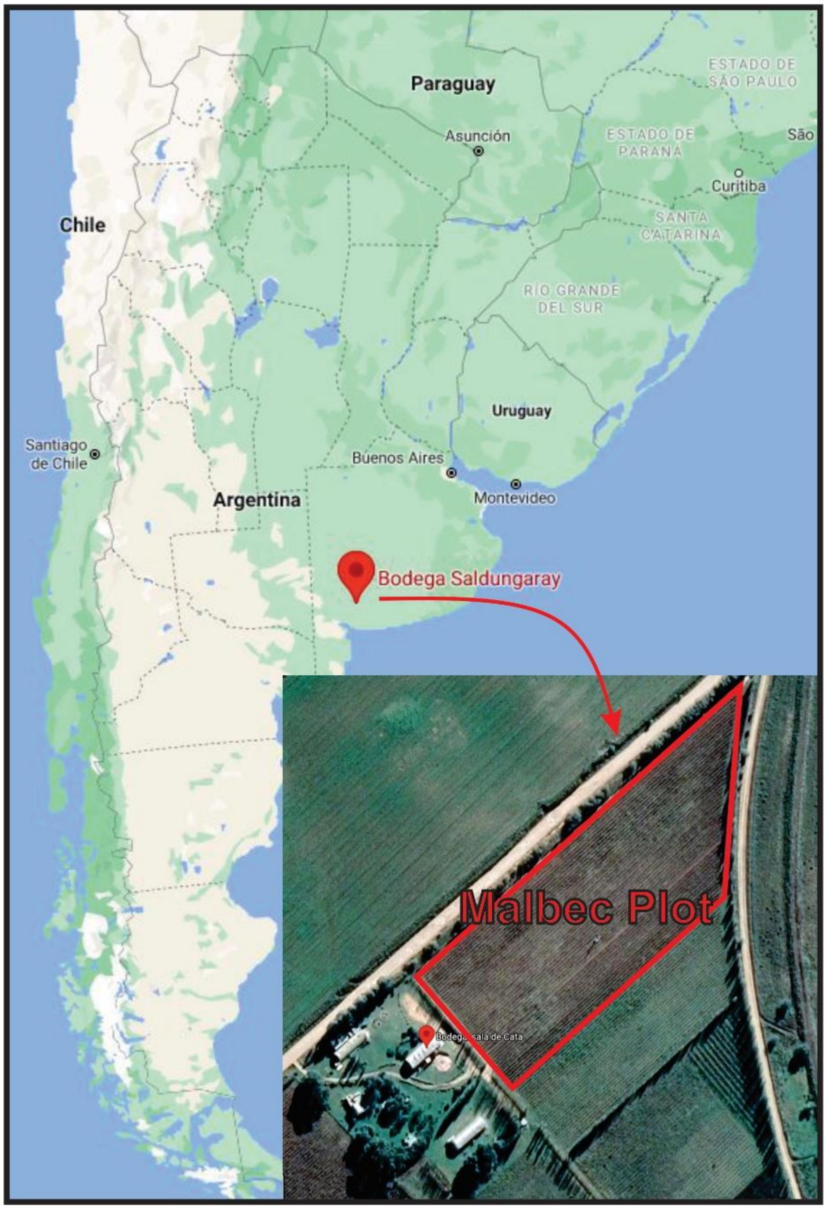

Supplementary figure 1: Saldungaray winery and vineyard, Saldungaray, Buenos Aires province, Argentina. Sample collection plot (Malbec varietal) is indicated with a red polygon. The imaged was produced by the authors using map data obtained from Google (map data ©2021 Google).

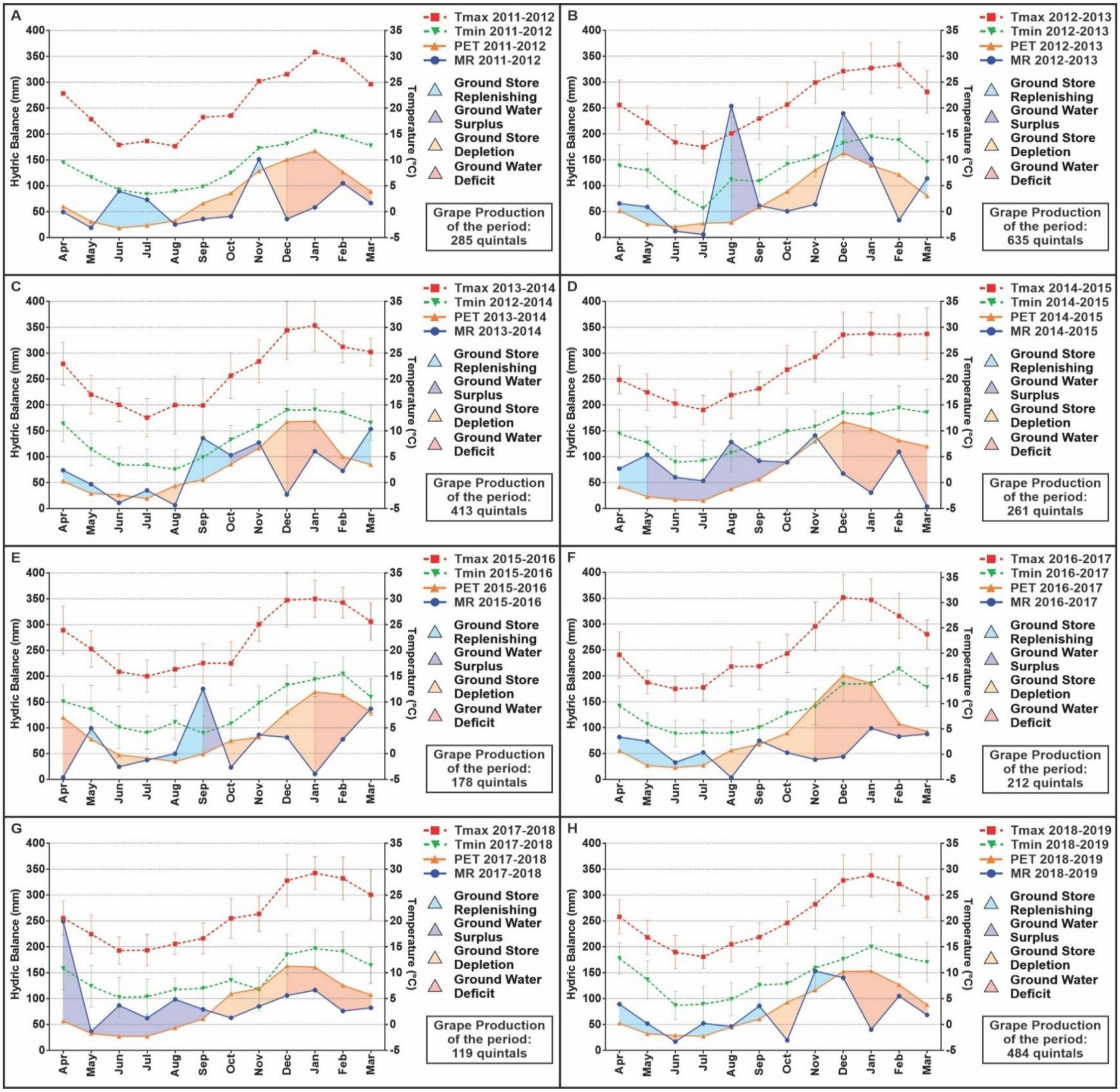

Supplementary figure 2: Climatic conditions in the years 2011 to 2019 (minimum and maximum temperatures and hydrological balances). Grape production is given for each vintage.

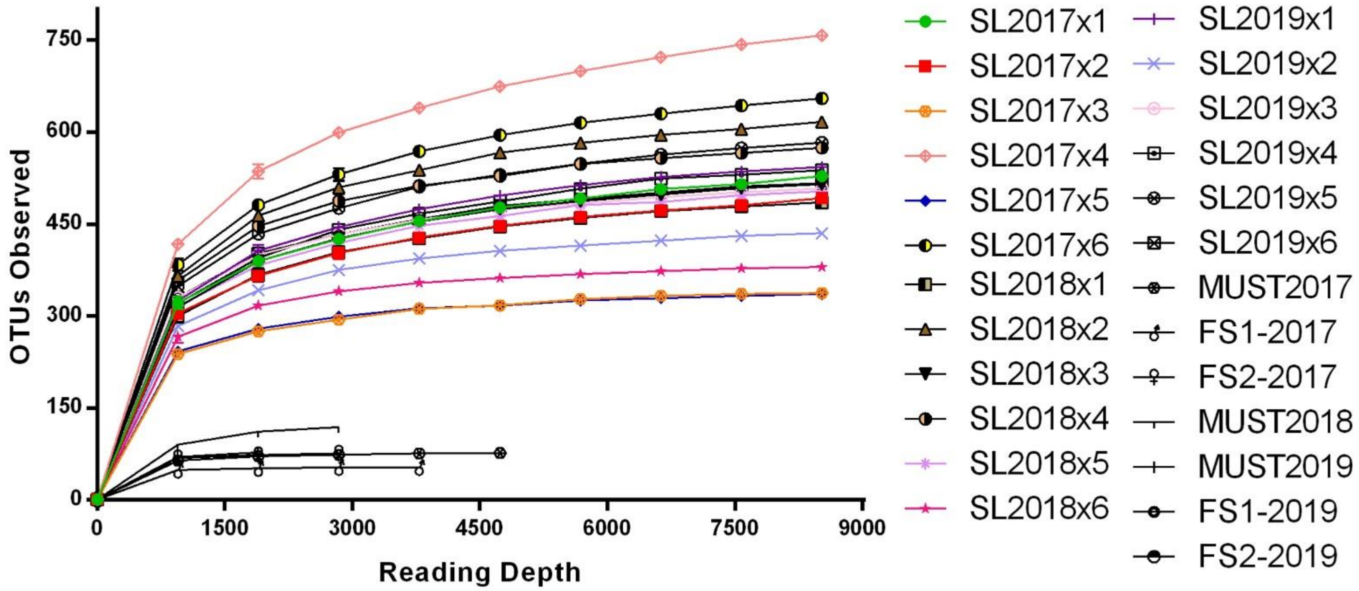

Supplementary figure 3: Bacterial rarefaction curves showing the OTUs observed in each sample and for the 3 vintages analyzed (2017, 2018, and 2019).

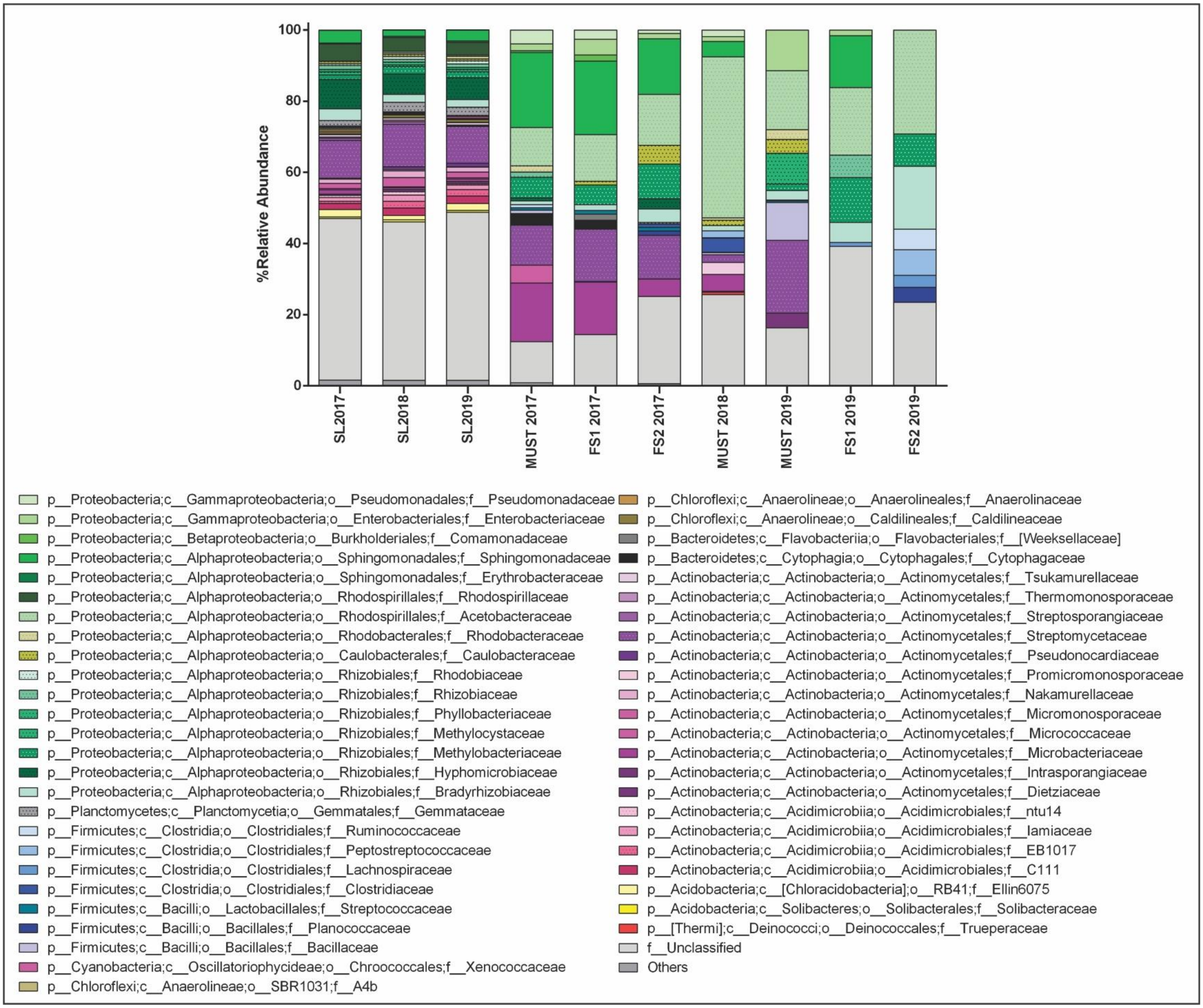

Supplementary figure 4: Microbial structure of the bacterial community for the soil (SL), and wine samples from the Saldungaray vineyard, at the family level. Only OTUs exhibiting a relative abundance ≥0.5% are shown. In addition, the “others” category includes minority (<0.5%) OTUs.

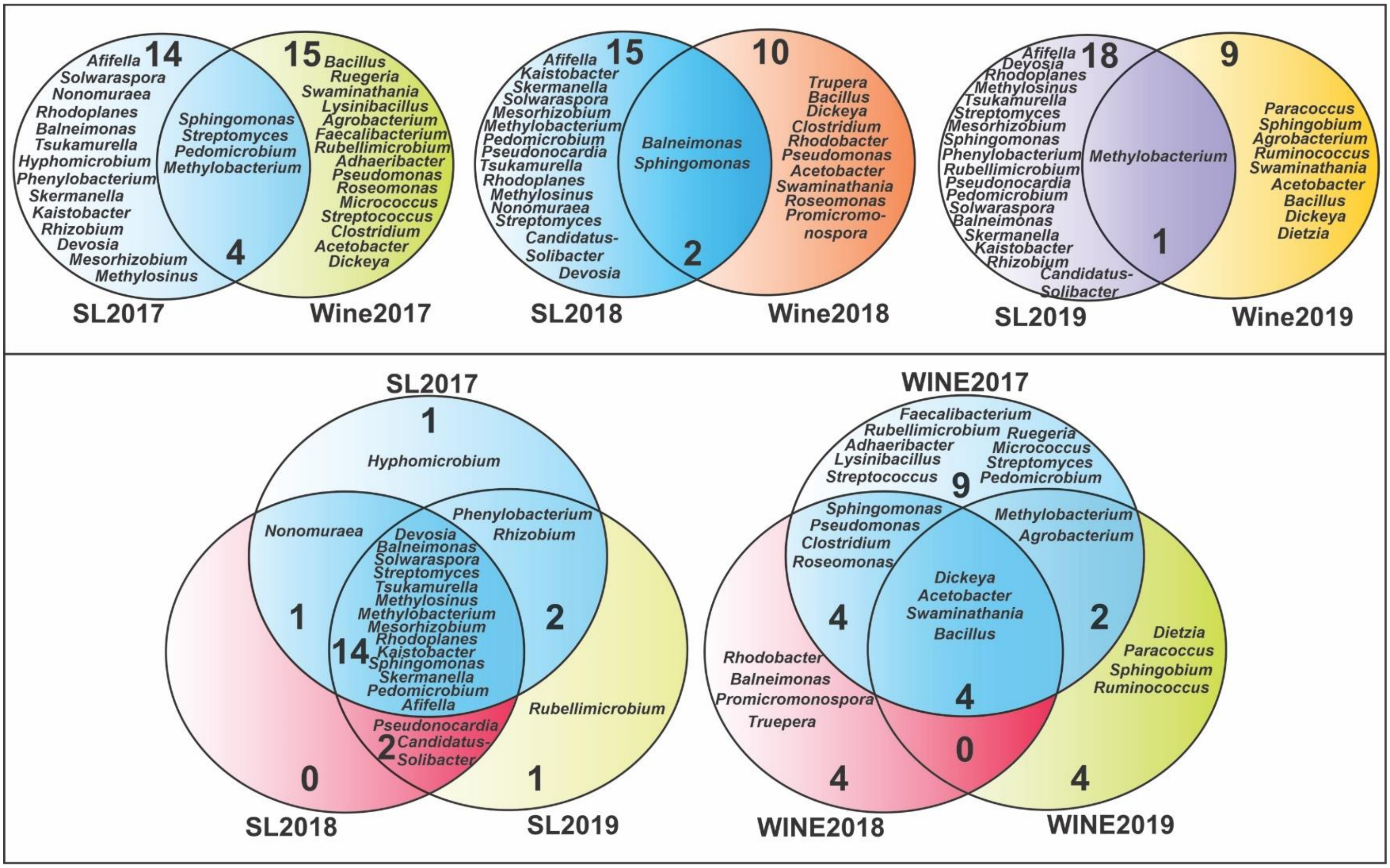

Supplementary figure 5: Venn diagram analysis for the bacterial diversity of Saldungaray vineyard at genus level. Soil-wine diversity relation **(A)**, soil-related diversity, and wine-related diversity according to vintage year **(B)**.
